# Effects of sleeve gastrectomy on bone mass, microstructure of femurs and bone metabolism associated serum factors in obese rats

**DOI:** 10.1186/s12902-021-00843-1

**Published:** 2021-08-26

**Authors:** Ying Xue, Ran Li, Yong Zhao, Ling Li, Yun Zhou

**Affiliations:** Department of Endocrinology and Metabolism, Tongji Hospital, School of Medicine, Tongji University, No. 389, Xincun Road, Shanghai, 200065 China

**Keywords:** Sleeve gastrectomy (SG), Bone metabolism, Adipokines, Protein tyrosine phosphatase 1B (PTP1B)

## Abstract

**Background:**

Sleeve gastrectomy (SG) is a profoundly effective operation for severe obese patients, but is closely associated with bone mass loss. Previous studies have reported changes of various serum factors which may be associated with bone mass loss after SG. However, those results are contradictory. In this study, we assessed the effects of SG on bone mass, microstructure of femurs, and changes in bone turnover markers (BTMs), serum adipokines, inflammatory factors and gastrointestinal hormones after SG in high-fat diet (HFD) induced obese rats.

**Methods:**

Eight-week-old male Sprague–Dawley (SD) rats were fed with HFD to induce obesity. Then, SG and sham surgery were performed in anesthetized obese rats. SD rats in control group were fed with standard chow. Microstructure of femurs was scanned and analyzed by micro-computed tomography in control group, HFD sham group and HFD SG group. Serum inflammatory factors, adipokines markers, gastrointestinal hormones and BTMs were also measured.

**Results:**

Bone mineral density (BMD) of trabecular bone in both HFD sham group and HFD SG group were remarkably decreased compared with control group. All serum BTMs were significantly higher in HFD SG group than HFD sham group. In the meantime, serum levels of several important inflammatory factors, gastrointestinal hormones and adipokines such as tumor necrosis factor-α (TNF-α), interleukin (IL)-6, monocyte chemoattractant protein-1(MCP-1), ghrelin, insulin and leptin in HFD SG group were remarkably reduced compared with HFD sham group, whereas glucagon-like peptide-1 (GLP-1), adiponectin, fibroblast growth factor (FGF)-19 and FGF-21 were dramatically increased after SG. Protein tyrosine phosphatase 1B (PTP1B) was significantly increased in the HFD sham group than control group. Spearman’s correlation analysis indicated that serum osteocalcin (OC) and 25-hydroxy vitamin D_3_ (25(OH)D_3_) were positively correlated with BMD of trabecular bone, whereas serum PTP1B and TNF-α were negatively related to BMD of trabecular bone.

**Conclusions:**

SG aggravates bone mass loss and activates bone remodeling in obese rats. Levels of BTMs, adipokines, inflammatory factors, and gastrointestinal hormones could be affected by SG in obese rats. Serum PTP1B level might be associated with abnormal bone mass in obese rats.

**Supplementary Information:**

The online version contains supplementary material available at 10.1186/s12902-021-00843-1.

## Introduction

Obesity, mainly caused by unhealthy dietary habits and sedentary lifestyle, is considered to be a serious public health problem [1]. It is a pathological state of excessive adipose tissue accumulation, which increases the risks of multiple diseases, such as cardiovascular disease, osteoporosis and type 2 diabetes [2]. Previous studies have shown that obesity has a negative impact on bone, including increased bone differentiation, decreased bone mineral density (BMD), and increased risks of fractures [3, 4]. A variety of adipokines, pro-inflammatory cytokines and chemokines secreted by adipose tissue may be part of the main reasons for the increased risks of osteoporosis caused by obesity [5–10].

In recent years, sleeve gastrectomy (SG), a commonly performed bariatric procedure around the world, has been proven to be a promising operation for weight loss and treating obesity comorbidities in patients with severe obesity [11, 12]. SG is considered to be a restrictive procedure by removing approximately 80% of the lateral stomach. It could significantly reduce food intake [13], induce alterations of hormones and accelerate gastrointestinal transit [14]. It has been found in previous literature that various factors secreted by adipose tissue were changed after SG [8, 15–17]. Growing evidence also has shown that there was no significant improvement of bone metabolism in obese patients after SG. There were even adverse effects of SG on bone metabolism in obese patients, including elevated bone turnover markers (BTMs), reduced BMD and increased fracture risk [18, 19]. According to the clinical and basic studies, bone loss after SG may be influenced by multiple factors. Restrictive gastric volume leads to malabsorptive effect on vitamin D and calcium, further causes secondary hyperparathyroidism [20]. Several gastrointestinal hormones and adipocytokines were produced abnormally after SG, which have been proposed to be responsible for bone loss after bariatric surgery [17, 21–26].

Protein tyrosine phosphatase 1B (PTP1B), a signaling enzyme, is mainly distributed on intracellular membranes and expressed in all insulin-responsive tissues. It plays a negative role in the insulin signaling pathway by dephosphorylating leptin receptor and insulin receptor [27]. Knocking out of PTP1B in mice could contribute to improved insulin sensitivity and attenuated hyperglycemia in obese mice [28]. Song et al. [29] indicated that Roux-en-Y gastric bypass could down-regulate the expression level of PTP1B in liver and improve hepatic glucose metabolism in obese rats. Katherine Zee et al. [30] found that the protein tyrosine phosphatases (PTPs) had relevance to the development and maintenance of the skeleton. However, it is still unclear whether PTP1B could be affected by SG and whether PTP1B could play a role in the bone loss after SG.

Our purpose of this study was to evaluate the effects of SG on BMD and microstructure of femurs in obese rats, and to access the changes in bone metabolism associated serum factors such as BTMS, adipokines, inflammatory cytokines and gastrointestinal hormones after SG.

## Materials and methods

### Animal model

Eight-week-old male SD rats (*n* = 20) were purchased from Model Animal Research Center of Nanjing University, caged individually with temperature 21 ± 4 °C, humidity 55 ± 5% and 12 h light/dark cycles, and housed in Laboratory Animal Center at Tongji University Hubei Campus. Rats were randomly divided into 2 groups: Control group (*n* = 5) and high-fat diet (HFD) group (*n* = 15). Rats in control group were fed with a normal chow diet (containing 6% fat, Xietong Biotech, China) for 16 weeks. Rats in HFD group were fed with a high fat diet (containing 42% fat, Puluteng Biotech, China) for 16 weeks to induce obese. All rats were allowed to drink water freely. The obese rats were further randomly assigned to the HFD sham group (*n* = 5) and the HFD SG group (*n* = 10). The surgical procedures of SG were performed as described previously [19]. Briefly, the lateral 70–80% of the stomach, including the entire fundus, was removed longitudinally along with the greater curvature of the stomach, resulting in a sleeve shape of the residual stomach and a significantly reduced gastric volume. All rats were sacrificed 4 weeks after SG surgery. Body weight and food intake were measured before and 4 weeks after surgery.

### Collection and measurement of blood samples

All rats were fasted for at least 12 h, then the blood samples were collected and stored at − 80 °C until measured. Fasting blood glucose (FBG) levels were measured using a hand-held glucometer (Johnson & Johnson Medical Ltd., USA). The levels of blood lipids, liver function, adipokines, inflammatory cytokines, BTMs and gastrointestinal hormones were tested using an enzyme-linked immunosorbent assay (ELISA) kit (Lai Er Bio-Tech, China), including total cholesterol (TC), triacylglycerol (TG), free fatty acid (FFA), alanine aminotransferase (ALT), aspartate aminotransferase (AST), leptin, adiponectin, angiopoietin-like protein 2 (ANGPTL2), suppressor of cytokine signalling-3 (SOCS3), PTP1B, and leukocyte cell-derived chemotaxin 2 (LECT2), interleukin (IL)-6, tumor necrosis factor-α (TNF-α), monocyte chemoattractant protein-1(MCP-1) and IL-1β, N-terminal propeptide of type I procollagen (PINP), N-terminal cross-linking telopeptide of type I collagen (NTX), bone specific alkaline phosphatase (BALP), C-terminal cross-linking telopeptide of type I collagen (CTX), osteocalcin (OC), 25-hydroxy vitamin D_3_ (25(OH)D_3_), parathyroid hormone (PTH), insulin, GLP-1, ghrelin, fibroblast growth factor (FGF)-21 and FGF-19. All procedures were performed in accordance with the manufacturer’s instructions. Homeostasis model assessment of insulin resistance (HOMA-IR) was calculated according to the following formula: HOMA-IR = (fasting insulin [mIU/L] × fasting glucose [mmol/L])/22.5.

### Microstructural bone analysis using micro-computed tomography (micro-CT)

Microstructure of rat femurs was scanned via micro-CT (SkyScan 1176, Bruker, Kontich, Belgium) following the procedures of Wong et al. [31]. The result of micro-CT was a two-dimensional (2D) cross sectional image in gray scale. Then, the 2D images obtained were reconstructed to three-dimensional (3D) models using NRecon software (version 1.6, SkyScan, Belgium). After the reconstruction images were obtained, the bone tissue was scanned and analyzed using CTAn software (1.13 version). The cortical bone was assessed as the following parameters: cortical percent bone volume (bone volume/ total volume, Ct.BV/TV), cortical bone surface density (Ct.BS/TV), cortical thickness (Ct.Th), cortical number (Ct.N), and standard deviation of trabecular thickness (CtTh.SD). Cancellous bone outcomes included trabecular percent bone volume (Tb. BV/TV), trabecular bone surface density (Tb.BS/TV), trabecular thickness (Tb.Th), trabecular number (Tb.N), trabecular separation (Tb.Sp), standard deviation of trabecular separation (TbSp.SD), standard deviation of trabecular thickness (TbTh.SD), trabecular pattern factor (Tb.Pf), connectivity density (Conn.Dens), structural model index (SMI), total porosity percent (To.Po), and euler number (Eu.N).

### Statistical analyses

Statistical analyses were performed using SPSS software version 22.0. All data were presented as mean ± standard deviation (SD), and analyzed by one-way ANOVA followed by the least significant differences (LSD) or Dunnett’s T3 post hoc multiple comparison test. A paired t test was used when comparing means amongst pre-operative and postoperative values in one group, whereas an independent Student’s t test was used to compare the means of two independent groups at the same time point. Correlation analysis was performed using Spearman’s test. All tests were two-tailed and the significant difference was set at *P* < 0.05.

## Results

### Effect of SG surgery on body weight, blood glucose and blood lipid profiles

**Fig. 1:**
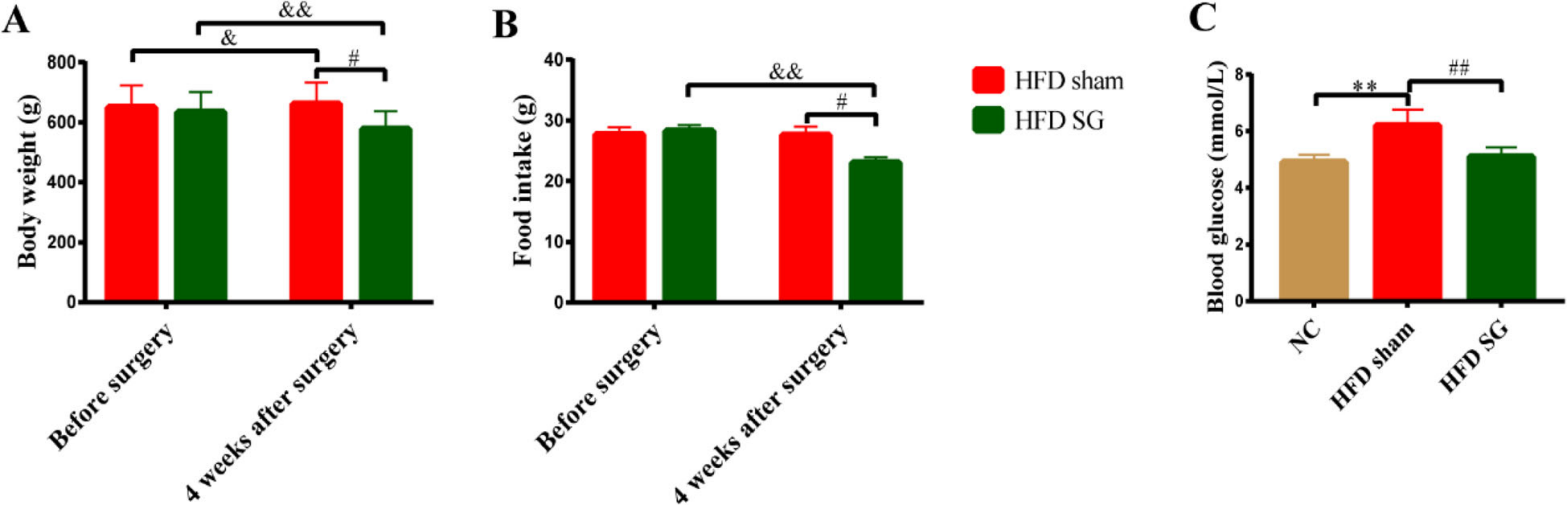
Body weight, food intake and fasting blood glucose of rats among three groups. (A-B) Changes in body weight and food intake of rats in HFD sham group and HFD SG group before and 4 weeks after surgery. (C) Changes in fasting blood glucose of rats among the control group, HFD sham group and HFD SG group at 4 weeks after surgery. Data are expressed as mean ± SD, control group *n* = 5, HFD sham group *n* = 5, HFD SG group *n* = 9, ^*^P<0.05, ^**^P<0.01 vs. Control group; ^#^*P*<0.05, ^##^*P*<0.01 vs. HFD sham group; ^&^*P*<0.05, ^&&^*P*<0.01 vs. before surgery

The body weight of rats in HFD sham group at 4 weeks after surgery was significantly higher than that before surgery. There was no significant difference in food intake of rats in HFD sham group between pre-operation and 4 weeks after surgery. However, the body weight and food intake of rats in HFD SG group at 4 weeks after SG were both obviously lower than those before SG and HFD sham group at 4 weeks after surgery (Fig. 1A, B). The FBG level of HFD sham group was significantly increased compared with the control group, while the FBG of HFD SG group was dramatically lower than that of HFD sham group (Fig. 1C). In addition, blood lipid profiles and liver functions of obese rats after SG surgery were significantly improved compared with HFD sham group (Supplementary Table 1).

### Effect of SG surgery on BMD

**Fig. 2:**
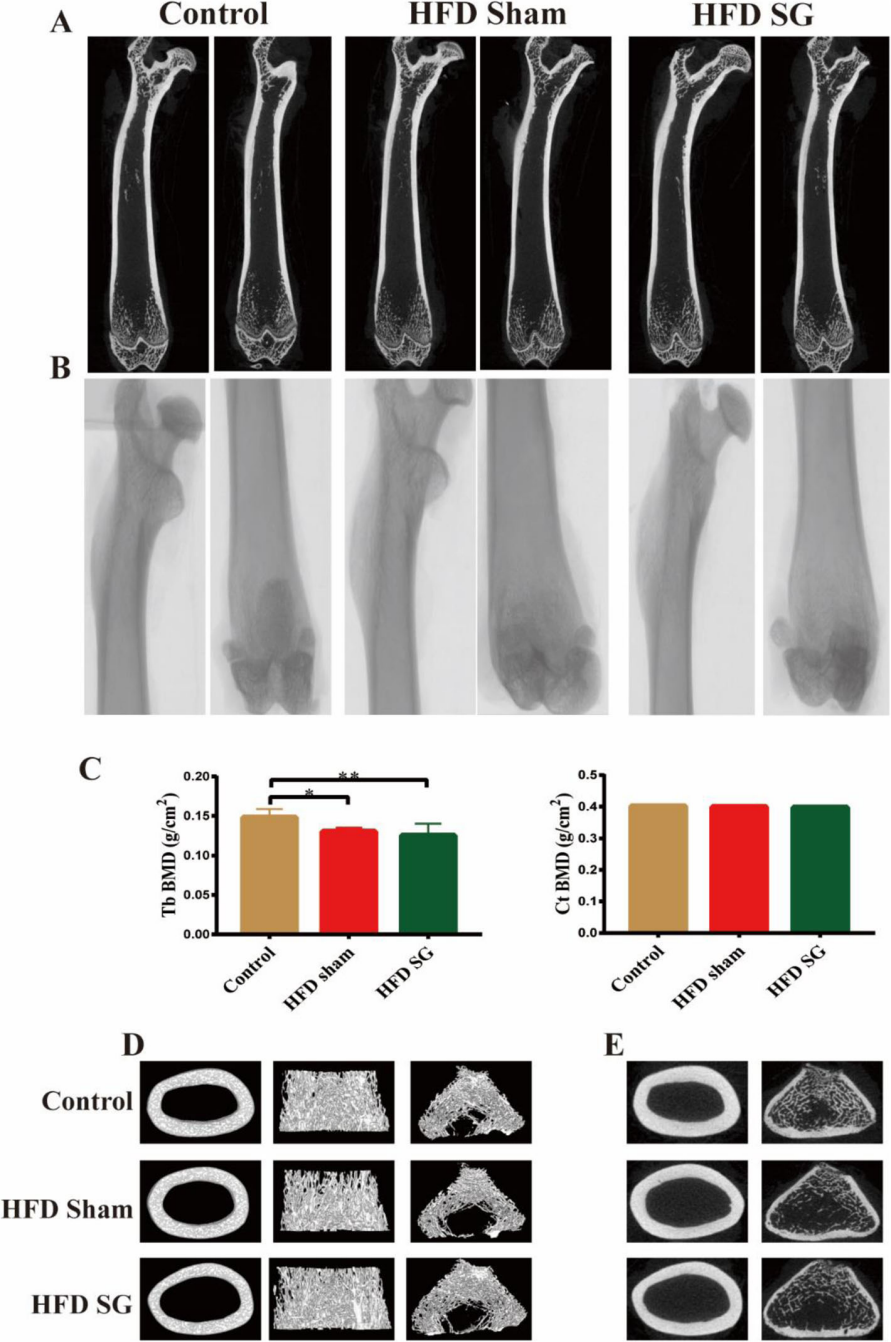
BMD and images of femoral bone microstructure examined by micro-CT among three groups. (A) 2D images of the longitudinal views at femur. (B) Unreconstructed images of the longitudinal views at femur. (C) BMD of trabecular and cortical bone. (D) 3D images of cortical bone at femur, trabecular bone at femur, and trabecular bone at distal femoral metaphysis respectively. (E) Unreconstructed images of the trabecular and cortical bones at femur. Data are expressed as mean ± SD, control group *n* = 5, HFD sham group *n* = 5, HFD SG group *n* = 9, ^*^P<0.05, ^**^P<0.01 vs. Control group. Tb BMD: the trabecular bone mineral density; Ct BMD: cortical bone mineral density

The trabecular BMD (Tb BMD) in both HFD sham group and HFD SG group were remarkably decreased compared with control group. Although there was no significant change in Tb BMD between HFD sham group and HFD SG group, a decrease in value still occur in HFD SG group, indicating a trend of further bone loss in trabecular bone after SG. In addition, there was no difference in BMD of cortical bone (Ct BMD) among control group, HFD sham group and HFD SG group (Fig. 2A, B, C).

### Effect of SG on bone microstructure

The 2D images of the longitudinal views at femur were showed in Fig. 2A. The 3D images of trabecular and cortical bones at femur, and trabecular at distal femoral metaphysis were illustrated in Fig. 2D. Unreconstructed images of the longitudinal views at femur, and the transverse views at femur of trabecular and cortical bones were demonstrated in Fig. 2B and E respectively.

The trabecular bone microarchitecture of femur was shown in Table 1. Compared to control group, Tb.BV/TV, Tb.BS/TV, and Tb. N in HFD sham group were significantly decreased, indicating trabecular bone loss in obesity rats. Although Tb. Th and TbTh.SD did not show significant difference between control group and HFD sham group, a downward trend still occur in HFD sham group. Tb.BV/TV, Tb.BS/TV, Tb. N and Tb. Th in SG operated rats were remarkably lower than control group as well. Although there was no significant difference in Tb.BV/TV, TbTh.SD and Tb. Th between HFD SG group and HFD sham group, a downward trend can still be seen in HFD SG group, suggesting a further reduction in trabecular bone after SG. Furthermore, compared with control group, increased Tb. Pf and To. Po in both HFD SG group and HFD sham group, raised levels of Tb. Sp in HFD SG group, and reduced Conn. Dens in HFD sham group demonstrated remarkable deterioration of trabecular connectivity due to the reduced number of bone trabecula. SMI was significantly higher in HFD SG groups than the control group, indicating that rod-like trabeculae gradually replaced plate-like trabeculae after SG. However, Eu. N did not differ among the three groups.

**Table 1:** The bone microstructure parameters in femur among three groups

	Control	HFD sham	HFD SG
**Cortical Bone Microstructure**			
Ct.BV/TV (%)	91.37 ± 0.28	91.50 ± 0.13	90.94 ± 0.29^*##^
Ct.BS/TV (mm^−1^)	4.50 ± 0.08	4.54 ± 0.05	4.76 ± 0.13^**##^
Ct.N (mm^−1^)	1.23 ± 0.03	1.26 ± 0.01	1.36 ± 0.05^**##^
Ct.Th (mm)	0.74 ± 0.02	0.73 ± 0.01	0.67 ± 0.03^**##^
CtTh.SD (mm)	0.125 ± 0.006	0.119 ± 0.005	0.110 ± 0.004^**#^
**Trabecular Bone Microstructure**			
Tb.BV/TV (%)	28.29 ± 8.09	18.75 ± 3.53^*^	17.94 ± 2.91^*^
Tb.BS/TV (mm^−1^)	7.06 ± 1.30	5.10 ± 0.89^*^	5.27 ± 0.70^*^
Tb.N (mm-1)	1.99 ± 0.42	1.40 ± 0.24^*^	1.44 ± 0.22^*^
Tb.Th (mm)	0.14 ± 0.01	0.13 ± 0.01	0.12 ± 0.01^*^
TbTh.SD (mm)	0.046 ± 0.004	0.044 ± 0.006	0.040 ± 0.002
Tb.Sp (mm)	0.45 ± 0.10	0.68 ± 0.20	0.82 ± 0.25^*^
TbSp.SD	0.32 ± 0.09	0.47 ± 0.19	0.69 ± 0.25
Tb.Pf (mm^−1^)	3.58 ± 2.52	6.15 ± 0.57^*^	6.60 ± 1.51^*^
SMI	1.44 ± 0.21	1.63 ± 0.07	1.72 ± 0.15^*^
To.Po (%)	71.71 ± 8.09	81.25 ± 3.53^*^	82.05 ± 2.91^*^
Eu.N	− 1413.20 ± 376.35	− 1169.20 ± 421.24	− 1484.00 ± 288.52
Conn.Dens (mm^−3^)	43.65 ± 10.40	27.86 ± 6.97^*^	32.17 ± 6.35

Data are expressed as mean ± SD, control group *n* = 5, HFD sham group *n* = 5, HFD SG group *n* = 9, ^*^P<0.05, ^**^P<0.01 vs. Control group; ^#^*P*<0.05, ^##^*P*<0.01 vs. HFD sham group

Ct.BV/TV: Cortical percent bone volume; Ct.BS/TV: Cortical bone surface density; Ct.N: Cortical number; Ct.Th: Cortical thickness; CtTh.SD: Standard deviation of cortical thickness; Tb. BV/TV: Trabecular percent bone volume; Tb.BS/TV: Trabecular bone surface density; Tb.N: Trabecular number; Tb.Th: Trabecular thickness; TbTh.SD: Standard deviation of trabecular thickness; Tb.Sp: Trabecular separation; TbSp.SD: Standard deviation of trabecular separation; Tb.Pf: Trabecular pattern factor; SMI: Structural model index; To.Po: Total porosity percent; Eu.N: Euler number; Conn.Dens: Connectivity density

For cortical parameters, Ct.BV/TV, Ct. Th and CtTh.SD were markedly lower in HFD SG group than both control group and HFD sham group. Ct.BS/TV and Ct. N were significantly increased in the HFD SG group compared to control group and HFD sham group, indicating cortical bone loss in obesity rats.

### Effect of SG on serum BTMs

**Fig. 3:**
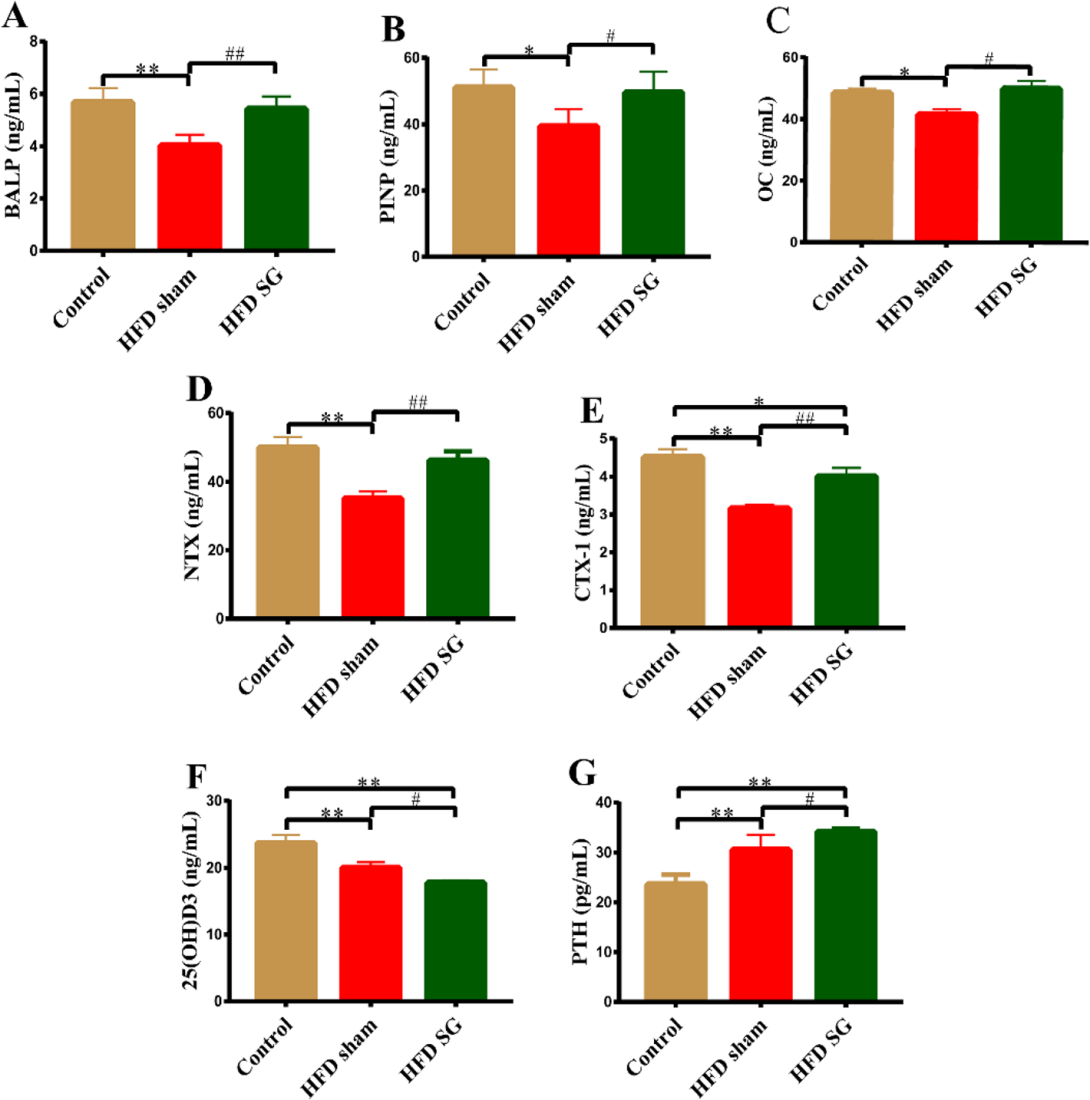
Alterations of BTMs among the three groups. Serum concentrations of (A) BALP, (B) PINP, (C) OC, (D) NTX, (E) CTX-1, (F) 25(OH)D3 and (G) PTH were evaluated by ELISA. Data are expressed as mean ± SD, control group *n* = 5, HFD sham group *n* = 5, HFD SG group *n* = 9, ^*^P<0.05, ^**^P<0.01 vs. Control group; ^#^*P*<0.05, ^##^*P*<0.01 vs. HFD sham group. BALP: Bone specific alkaline phosphatase; PINP: N-terminal propeptide of type I procollagen; OC: Osteocalcin; NTX: N-terminal cross-linking telopeptide of type I collagen; CTX-1: C-terminal cross-linking telopeptide of type I collagen; 25(OH)D3: 25-hydroxy vitamin D3; PTH: Parathyroid hormone

The values of bone formation marker (such as PINP, BALP and OC), and bone resorption marker (such as CTX-1and NTX) were all significantly decreased in the obese rats of HFD sham group compared to the control group, whereas the above variables in HFD sham groups were all significantly increased after SG. Our data indicated the bone turnover rate was increased significantly after SG surgery. Serum PTH levels rose dramatically in both HFD sham group and HFD SG group compared with controls, while PTH level in HFD SG group was remarkably higher than HFD sham group. We speculate that the increased PTH levels after SG might be due to the negative feedback of decreased serum 25(OH)D_3_ levels in HFD SG group compared with HFD sham group (Fig. 3).

### Effect of SG surgery on gastrointestinal hormones and adipocytokines

**Fig. 4:**
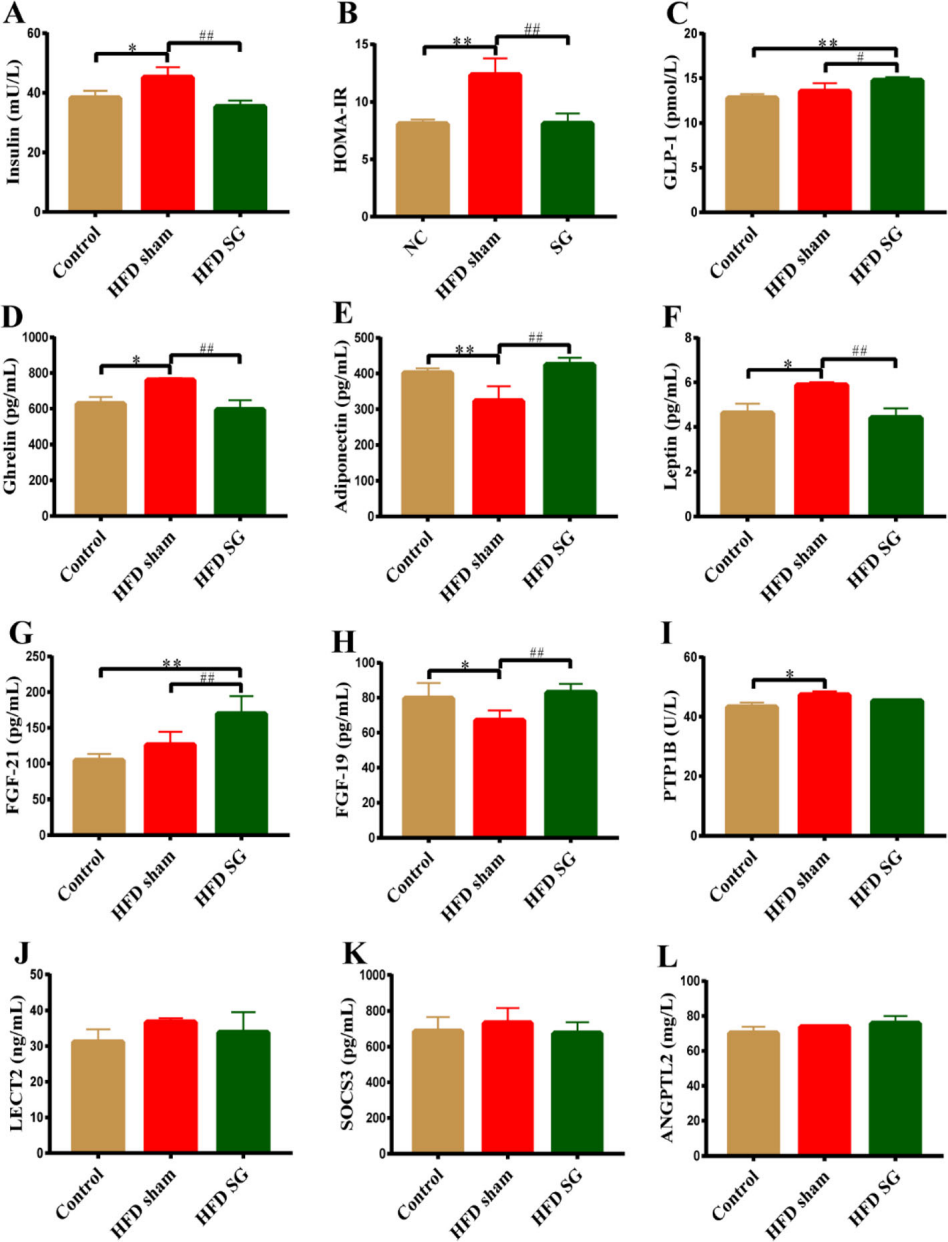
Changes in gastrointestinal hormone, adipocytokines among three groups. Serum levels of (A) Insulin, (B) HOMA-IR, (C) GLP-1, (D) Ghrelin, (E) Adiponectin, (F) Leptin, (G) FGF-21, (H) FGF-19, (I) PTP1B, (J) LECT2, (K) SOCS3 and (L) ANGPTL2 were measured by ELISA. Data are expressed as mean ± SD, control group *n* = 5, HFD sham group *n* = 5, HFD SG group *n* = 9, ^*^P<0.05, ^**^P<0.01 vs. Control group; ^#^*P*<0.05, ^##^*P*<0.01 vs. HFD sham group. GLP-1: Glucagon-like peptide-1; FGF: Fibroblast growth factor; PTP1B: Protein tyrosine phosphatase 1B; LECT2: Leukocyte cell-derived chemotaxin 2; SOCS3: Suppressor of cytokine signalling-3; ANGPTL2: Angiopoietin-like protein 2

We detected the levels of several representative gastrointestinal hormones and crucial adipokines after SG in obese rats. As shown in Fig. 4A-4L, concentrations of serum insulin and ghrelin, and HOMA-IR values were significantly higher in the HFD sham group than those in the control group, whereas those indicators were all markedly decreased after SG. In contrast, GLP-1 was dramatically increased in HFD SG group than HFD sham group. Therefore, we speculate appetite suppression and hyperinsulinemia improvement in obese rats after SG may be partly due to the alterations of the above gastrointestinal hormones. In addition, serum leptin and PTP1B were significantly increased in the HFD sham group than control group, while leptin was notably lower after SG than HFD sham group. However, there was no significant difference in PTP1B between HFD sham group and HFD SG group. Conversely, serum adiponectin, FGF19 and FGF-21 were significantly increased after SG compared to HFD sham group. There was no statistically difference in LECT2, SOCS3 and ANGPTL2 among the three groups.

### Effect of SG surgery on inflammatory cytokines

**Fig. 5:**
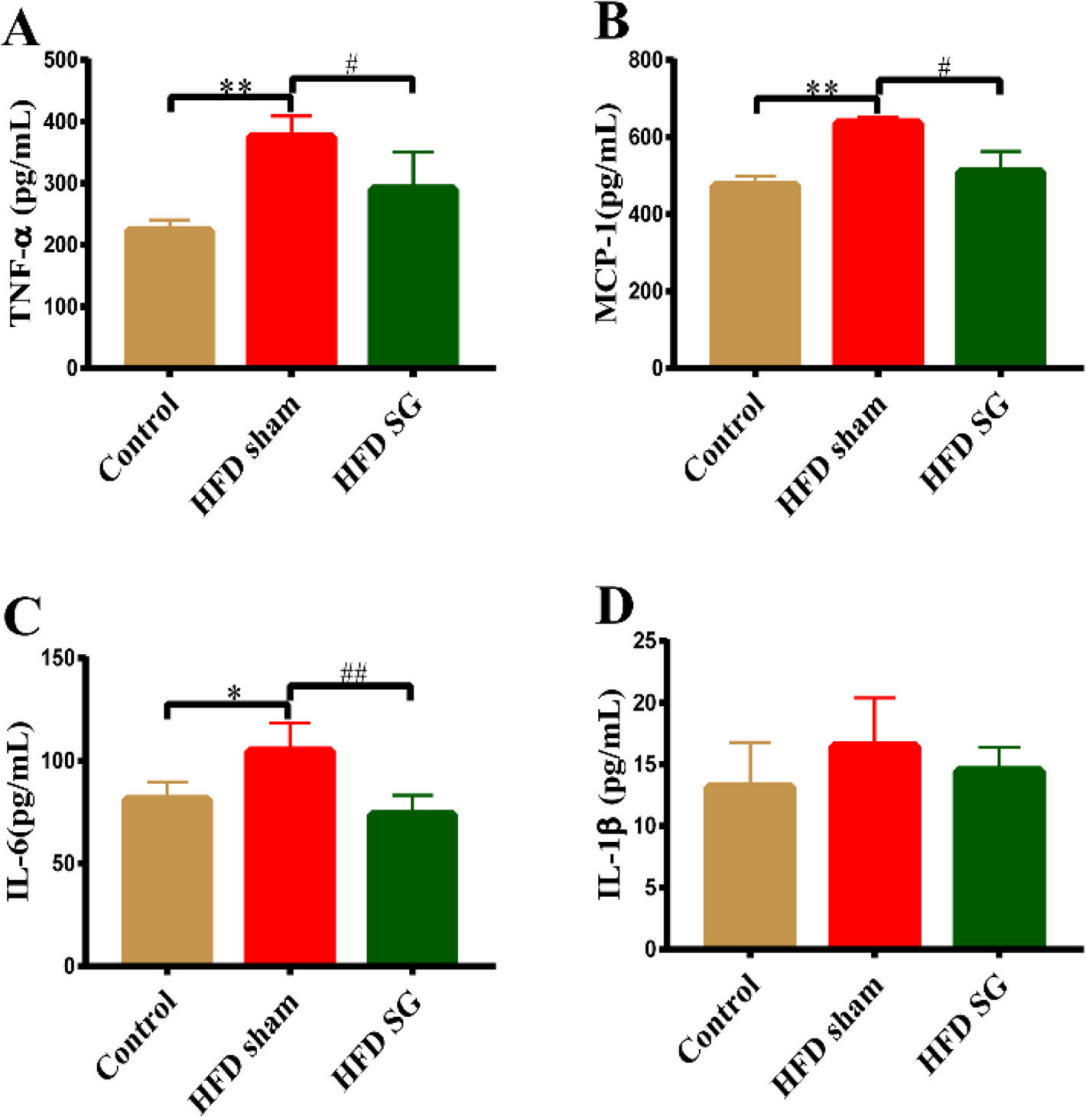
Changes in inflammatory cytokines among three groups. (A-D) Serum levels of TNF-α, MCP-1, IL-6, and IL-1β were measured by ELISA. Data are expressed as mean ± SD, control group *n* = 5, HFD sham group *n* = 5, HFD SG group *n* = 9, ^*^P<0.05, ^**^P<0.01 vs. Control group; ^#^*P*<0.05, ^##^*P*<0.01 vs. HFD sham group. TNF-α: Tumor necrosis factor-α; MCP-1: Monocyte chemoattractant protein-1; IL-6: Interleukin-6; IL-1β: Interleukin-1β

To explore the impact of SG surgery on inflammatory cytokines, we tested the levels of four important inflammatory factors including TNF-α, MCP-1, IL-6 and IL-1β in three groups. Serum TNF-α, MCP-1 and IL-6 were significantly increased in the HFD group than control group, whereas those inflammatory factors were all dramatically lower after SG than HFD sham group. There was no significantly difference in IL-1β among the three groups (Fig. 5).

### Serum PTP1B was negatively correlated with BMD in trabecular bones

Spearman’s correlation analysis was shown in Table 2. Serum OC and 25(OH)D3 were positively correlated with Tb BMD, whereas serum PTP1B and TNF-α were negatively related to Tb BMD. However, there were no association between other serum indices and BMD (Tb BMD and Ct BMD). We further analyzed the correlation between serum PTP1B and bone microstructure parameters. However, we didn’t find any correlation between serum PTP1B and bone microstructure parameters (Table 3).

**Table 2:** Spearman’s correlations between BMD and serum variables, including biochemical indexes, adipokines, inflammatory cytokines, BTMs and gastrointestinal hormones

Parameter		Tb BMD	Ct BMD
PINP	*R*	0.067	− 0.527
	*P*	0.855	0.117
BALP	*R*	0.317	0.117
	*P*	0.406	0.765
OC	*R*	0.900*	0.100
	*P*	0.037	0.873
NTX	*R*	0.631	0.703
	*P*	0.129	0.078
CTX-1	*R*	−0.607	0.071
	*P*	0.148	0.879
25(OH)D3	*R*	0.762*	0.524
	*P*	0.028	0.183
PTH	*R*	−0.444	− 0.176
	*P*	0.232	0.651
Insulin	*R*	0.071	0.452
	*P*	0.867	0.260
GLP-1	*R*	−0.048	0.286
	*P*	0.911	0.493
ghrelin	*R*	0.143	0.214
	*P*	0.760	0.645
Adiponectin	*R*	−0.036	−0.143
	*P*	0.939	0.760
Leptin	*R*	0.048	0.190
	*P*	0.911	0.651
FGF-21	*R*	−0.500	0.067
	*P*	0.170	0.865
FGF-19	*R*	−0.150	−0.433
	*P*	0.700	0.244
PTP1B	*R*	−0.821*	0.036
	*P*	0.023	0.939
LECT2	*R*	−0.545	−0.509
	*P*	0.083	0.110
SOCS-3	*R*	0.095	−0.167
	*P*	0.823	0.693
ANGPTL2	*R*	−0.450	−0.450
	*P*	0.310	0.310
TNF-α	*R*	−0.821	0.464
	*P*	0.023*	0.294
MCP-1	*R*	−0.607	−0.286
	*P*	0.148	0.535
IL-6	*R*	−0.287	0.036
	*P*	0.490	0.933
IL-1β	*R*	−0.600	−0.257
	*P*	0.208	0.623
TC	*R*	0.311	0.098
	*P*	0.382	0.789
TG	*R*	−0.381	−0.119
	*P*	0.352	0.779
FFA	*R*	0.108	0.144
	*P*	0.818	0.758
ALT	*R*	−0.029	0.429
	*P*	0.957	0.397
AST	*R*	−0.048	0.262
	*P*	0.911	0.531

Control group *n* = 5, HFD sham group *n* = 5, HFD SG group *n* = 9, ^*^P<0.05

Tb BMD: the trabecular bone mineral density; Ct BMD: cortical bone mineral density; PINP: N-terminal propeptide of type I procollagen; BALP: Bone specific alkaline phosphatase; OC: Osteocalcin; NTX: N-terminal cross-linking telopeptide of type I collagen; CTX-1: C-terminal cross-linking telopeptide of type I collagen; 25(OH)D3: 25-hydroxy vitamin D3; PTH: Parathyroid hormone; GLP-1: Glucagon-like peptide-1; FGF: Fibroblast growth factor; PTP1B: Protein tyrosine phosphatase 1B; LECT2: Leukocyte cell-derived chemotaxin 2; SOCS3: Suppressor of cytokine signalling-3; ANGPTL2: Angiopoietin-like protein 2; TNF-α: Tumor necrosis factor-α; MCP-1: Monocyte chemoattractant protein-1; IL-6: Interleukin-6; IL-1β: Interleukin-1β; TC: Total cholesterol; TG: Triacylglycerol; FFA: Free fatty acid; ALT: Alanine aminotransferase; AST: Aspartate aminotransferase

**Table 3:** Spearman’s correlations between serum PTP1B and bone microstructure parameters in trabecular and cortical bones

PTP1B		BV/TV	BS/TV	Th	Sp	N	Pf	SMI	To.Po	Eu.N	Conn.Dens	Th.SD	Sp.SD
Tb	*R*	−0.607	−.0643	−0.464	0.500	−0.714	0.750	−0.200	0.607	0.250	−0.714	−0.536	0.393
	*P*	0.148	0.119	0.294	0.253	0.071	0.052	0.747	0.148	0.589	0.071	0.215	0.383
Ct	*R*	0.179	0.071	−0.179	–	0.179	–	–	–	–	–	−.429	–
	*P*	0.702	0.879	0.702	–	0.702	–	–	–	–	–	.337	–

Control group *n* = 5, HFD sham group *n* = 5, HFD SG group *n* = 9, * *P* < 0.05

PTP1B: Protein tyrosine phosphatase 1B; Tb: Trabecular; Ct: Cortical; BV/TV: Percent bone volume; BS/TV: Bone surface density; Th: thickness; Sp: Separation; N: Number; Pf: Pattern factor; SMI: Structural model index; To.Po: Total porosity percent; Eu.N: Euler number; Conn.Dens: Connectivity density; Th.SD: Standard deviation of thickness; Sp.SD: Standard deviation of separation

## Discussion

Bariatric surgery, especially SG, is an effective procedure for many severe obese individuals, including those suffering from type 2 diabetes [11, 12]. In the present study, we performed SG on rat models of HFD-induced obesity and confirmed favorable metabolic changes, namely, significant improvements in blood glucose, serum lipids and liver functions. Meanwhile, the significant decrease in pro-inflammatory adipokines such as leptin, TNF-α, IL-6 and MCP-1, and increase in anti-inflammatory adipokines such as adiponectin in HFD SG group compared to HFD sham group were found in our study, which might be beneficial for weight loss and improvement of hyperinsulinemia after SG in obese rats. Those data were consistent with previous studies of SG [32, 33].

In contrast to the above comparable benefits, our study found that SG did not improve the decreased BMD, deteriorated bone microstructure and abnormal bone remodeling in rats with HFD-induced obesity. Previous study has shown that micro-CT is very favorable for bone imaging because of high effective atomic weight of bone and the natural contrast between bone and soft tissue. Indicated micro-CT can also display both 2D cross-sectional bone images and 3D reconstructions of entire bones [34]. Trabecular bone microarchitecture and cortical bone morphology can be clearly visualized after the 3D reconstructions [34]. Our data demonstrated that obesity resulted in a significant loss of trabecular bone and a significant decrease in trabecular connectivity, which were primarily reflected in reduced Tb.BV/TV, Tb.BS/TV, Tb. N, Conn. Dens, and increased Tb. Pf, Tb. Po in HFD sham group compared with controls. Although there was no significant difference in all above parameters of trabecular bone between HFD sham group and HFD SG group, they still had an upward trend of Tb. Sp, TbSp.SD, Tb. Pf, SMI and Tb. Po, and a downward trend of Tb.BV/TV, TbTh.SD and Tb. Th after SG than HFD sham group, suggesting a further reduction in trabecular bone after SG. In addition, there was no substantial deterioration of cortical bone after SG in obese rats, due to no significant difference in the Ct.BMD and an significant increase in Ct.BS/TV and Ct. N after SG. Therefore, we found that the negative impact of SG on trabecular bone exceeds that on cortical bone, which might be due to the firmness and compactness of cortical bone.

BTMs are products of decomposition and synthesis of bone tissue itself, which can be detected in blood or urine. BTMs, including bone formation markers and bone resorption markers, are considered to manifest the activity of either osteoblasts or osteoclasts, thereby dynamically reflecting bone remodeling. Levels of PINP in serum reflects the ability of osteoblasts to synthesize bone collagen. BALP, an extracellular enzyme secreted by osteoblasts, is closely related to the mineralization of bone matrix. OC is the most abundant non-collagen protein in bone tissue and essential for the mineralization of bone matrix. Therefore, the above three markers which are involved in the process of bone formation are signs of osteoblast maturity and activity. CTX and NTX are the degradation products of bone collagen, reflecting the bone resorptive activity of osteoclasts, and are used as markers of bone resorption [19, 35]. We found that SG significantly increased serum concentrations of both bone formation marker (P1NP, BALP and OC), and bone resorption formation marker (CTX-1 and NTX), suggesting that SG can increase bone turnover ratio, thus leading to bone loss. In addition, we observed that there was a significant decrease in 25(OH)D3 and a notably increase in PTH levels in obese rats after SG compared to HFD sham group. We surmised that 25(OH)D3 deficiency and secondary increase in PTH after SG may aggravate bone turnover, further leading to bone loss and fracture [20].

In our study, in addition to weight gain, the serum insulin levels and HOMA-IR of obese rats induced by HFD were also significantly higher than those of the control group. Previous studies have found that obesity and insulin resistance affect bone homeostasis, leading to weakened bone formation and damaged bone structure [7, 36]. An in vivo study has shown that mice with insulin resistance exhibit enhanced bone marrow adipogenesis and decreased brown adipose tissue gene expression, suggesting that insulin resistance impairs bone anabolism [36]. Moreover, insulin signaling dysfunction in obesity caused by impaired binding of insulin-like growth factor-1 (IGF-1) to insulin receptors on osteoblasts also had a negative impact on bone remodeling [37, 38]. Our research also found that HFD-induced obesity led to bone loss and bone microstructure abnormalities in rats, which were consistent with previous studies. However, although obese rats after SG showed significant weight loss, decreased serum insulin levels and improved HOMA-IR, there was no improvement in bone mass and bone microstructure. This result suggested that the influence of SG on bone mass and bone microstructure is complex and multi-factorial. In addition to the insulin signaling pathway, bone mass and bone microstructure may also be affected by other important factors.

Our data demonstrated that SG also caused significant changes in the secretion of a variety of gastrointestinal hormones (such as ghrelin and GLP-1), which are directly or indirectly related to various bone regulation mechanisms. Ghrelin is a polypeptide that mainly synthesized in the fundus of the stomach and plays an important role in energy metabolism, food intake and gastric function [22]. Fukushima et al. [39] has reported that serum ghrelin may be produced by osteoblasts and could be positively correlated with trabecular BMD. GLP-1, releasing from the colon after eating, exerts an important influence on glycolipid metabolic and food intake [21]. Bernardo et al. [40] has showed that GLP-1 may facilitate bone formation deficiency and structural defects in rats with glucose intolerance. Our data demonstrated the decreased ghrelin and increased GLP-1 in obese rats after SG, which might be one reason for the bone loss after SG in obese rats.

In our study, serum FGF-19 level rose dramatically in obese rats after SG compared with HFD sham group, and serum FGF-21 was also increased significantly after SG. Haluzı’kova et al. [17] found serum FGF-19 level was increased nearly twice after SG in obese patients. That trend of FGF-19 in obese patients is similar to our data in obese rats. Previous studies have demonstrated the alterations of FGF-21 after SG, however, those results were variable. Two studies revealed that FGF-21 concentration was decreased after SG [17, 41]. Another study found FGF-21 level was increased at 6 or 12 months after SG [42]. Those discrepancies of FGF-21 level in our study and previous literature may be due to the different research objects and distinct observation duration after SG. Previous studies have suggested the potential functions of FGF-19 and FGF-21 in bone metabolism. FGF-19 may affect the skeletal system, and play a role in cartilage and bone growth [23]. FGF-21 was inversely associated with BMD [24, 43]. It may induce bone loss by increasing bone resorption and reducing bone formation [44]. Consequently, we hypothesized that bone loss after SG might partly be related to FGF-19 and FGF-21.

Our study also found serum leptin were significantly increased in the HFD sham group than control group, whereas it was notably lower after SG than HFD sham group. One study indicated that BMD was remarkably reduced in leptin-deficient mouse (ob/ob) [45], and leptin could directly promote osteoblasts and chondrocytes growth, further affect BMD [45]. We speculated SG could decrease leptin production in obese rats, and reduced leptin might play a part in the bone loss after SG. The hepatic expression of PTP1B was significantly decreased in obese rats after Roux-en-Y gastric bypass [29]. However, the impact of SG surgery on PTP1B and the relationship between PTP1B and BMD have not been illustrated yet. Our study found that serum PTP1B was significantly increased in obese rats compared with the controls, and PTP1B level was negatively correlated with Tb BMD. There was no significant change in serum PTP1B between HFD sham group and HFD SG group in obese rats. Our study suggested that PTP1B might be associated with bone metabolism and increased PTP1B might be related to the bone loss in obese rats. PTP1B, a member of PTPs family, is involved in regulating the level of tyrosine phosphorylation, and plays an important role in signal transduction pathways that control cell growth, differentiation and metabolism [46, 47]. One previous literature indicated the effect of PTP1B on osteoblasts [47]. V. Lezcano et al. found cytoplasmic PTP1B was highly expressed in ROS 17/2.8 osteoblasts (derived from rat osteosarcoma), and bisphosphonates could stimulate osteoblast proliferation by inhibiting cytoplasmic PTP1B [47]. Furthermore, previous studies have illustrated that PTP1B is a negative regulator of the leptin and insulin signaling pathways [48–51]. For leptin, it was reported that PTP1B deletion increased leptin sensitivity [51]. Zabolotny et al. [52] also indicated PTP1B could directly regulate leptin signaling in peripheral tissues. Their data demonstrated that mice with PTP1B-deficiency on a high-fat diet were hypersensitive to leptin, exhibiting reduced serum leptin levels. Further research demonstrated PTP1B negatively regulated leptin signaling via dephosphorylation of Jak2 [53]. In addition, Ahmad et al. [48] elucidated that PTP1B could directly act on the insulin receptor and play a role in the negative regulation of insulin signaling. Their results also elucidated that insulin signaling could be enhanced by the specific inhibition of PTP1B [48]. Another previous study has indicated that insulin receptor is a substrate of PTP1B in rodent osteoblasts. It was reported that PTP1B was able to interact directly with the endogenous β subunit of the insulin receptor in osteoblasts [50]. Therefore, we suggest that PTP1B may play a role in the bone metabolism via regulating leptin and insulin. However, further experiments are still needed to confirm the function of PTP1B in the regulation of bone metabolism and clarify molecular mechanism.

There are several limitations in our study. Firstly, the changes in adipokines, cytokines, gastrointestinal hormones and bone metabolism were not investigated in different time periods. Secondly, the mechanism of the relationship between those serum indicators and bone metabolism after SG has not been further explored. Thirdly, changes of sex hormones in obese rats were not detected in our study.

## Conclusions

In summary, SG operated on HFD-induced obese rats can activate bone remodeling, exacerbate the destruction of femoral bone microstructure, and ultimately lead to trabecular bone loss. Adipokines, inflammatory factors, gastrointestinal hormones may participate in the influence of SG surgery on bone loss. Our study found that serum PTP1B was negatively correlated with Tb BMD, suggesting PTP1B may be involved in the regulation of bone metabolism. Our study will be helpful to better understand the effects of SG on bone mass, microstructure of femurs and bone metabolism associated serum factors in obese patients.

## Supplementary Information

**Additional file 1 Supplementary Table 1**. Biochemical parameters were improved after SG in obese rats.

## Data Availability

All data and materials have been presented in the manuscript. No outliers were omitted from the main statistical analysis. Related information is available under request to the corresponding author.

## Abbreviations

SG: Sleeve gastrectomy
BTMs: Bone turnover markers
HFD: High-fat diet
SD: Sprague–Dawley
BMD: Bone mineral density
TNF-α: Tumor necrosis factor-α
IL-6: Interleukin-6
MCP-1: Monocyte chemoattractant protein-1
GLP-1: Glucagon-like peptide-1
FGF: Fibroblast growth factor
PTP1B: Protein tyrosine phosphatase 1B
OC: Osteocalcin
25(OH)D3: 25-hydroxy vitamin D3
IL-1β: Interleukin-1β
IGF-1: Insulin-like growth factor-1
SOCS3: Suppressor of cytokine signalling-3
LECT2: Leukocyte cell-derived chemotaxin 2
TC: Total cholesterol
TG: Triacylglycerol
FFA: Free fatty acid
ALT: Alanine aminotransferase
AST: Aspartate aminotransferase
ANGPTL2: Angiopoietin-like protein 2
PINP: N-terminal propeptide of type I procollagen
NTX: N-terminal cross-linking telopeptide of type I collagen
BALP: Bone specific alkaline phosphatase
CTX-1: C-terminal cross-linking telopeptide of type I collagen
PTH: Parathyroid hormone
micro-CT: Micro-computed tomography
2D: Two-dimensional
3D: Three-dimensional
Ct.BV/TV: Cortical percent bone volume
Ct.BS/TV: Cortical bone surface density
Ct.Th: Cortical thickness
Ct.N: Cortical number
CtTh.SD: Standard deviation of trabecular thickness
Tb. BV/TV: Trabecular percent bone volume
Tb.BS/TV: Trabecular bone surface density
Tb.Th: Trabecular thickness
Tb.N: Trabecular number
Tb.Sp: Trabecular separation
TbSp.SD: Standard deviation of trabecular separation
TbTh.SD: Standard deviation of trabecular thickness
Tb.Pf: Trabecular pattern factor
Conn.Dens: Connectivity density
SMI: Structural model index
To.Po: Total porosity percent
Eu.N: Euler number
LSD: Least significant differences
Tb BMD: Trabecular BMD
Ct BMD: Cortical BMD
PTPs: Protein tyrosine phosphatases
