# Supplementary material for: Effects of sleeve gastrectomy on bone mass, microstructure of femurs and bone metabolism associated serum factors in obese rats
Source: BMC Endocr Disord. 2021 Aug 26;21:173. doi: 10.1186/s12902-021-00843-1 (PMC8394165; doi:10.1186/s12902-021-00843-1)
Supplement: Supplementary file 1 — Additional file 1 Supplementary Table 1. Biochemical parameters were improved after SG in obese rats. [file 12902_2021_843_MOESM1_ESM.docx]

**Supplementary Table 1. Biochemical parameters were improved after SG in obese rats.**

|  | **Control** | **HFD sham** | **HFD SG** |
| --- | --- | --- | --- |
| TC (mmol/L) | 6.47±0.03 | 6.96±0.19**^*^** | 6.30±0.36^##^ |
| TG (mmol/L) | 1.48±0.25 | 2.50±0.13**^**^** | 1.79±0.28^##^ |
| FFA (μmol/L) | 520.57±137.60 | 818.80±130.50**^*^** | 482.79±95.86^#^ |
| ALT (U/L) | 107.61±7.20 | 127.17±11.14 | 95.61±10.80^##^ |
| AST(U/L) | 80.59±19.72 | 162.48±13.13**^**^** | 95.85±25.62^##^ |

Data are expressed as mean ± SD, control group *n* = 5, HFD sham group *n* = 5, HFD SG group *n* = 9, ^*^*P*＜0.05, ^**^*P*＜0.01 vs. Control group; ^#^*P*＜0.05, ^##^*P*＜0.01 vs. HFD sham group.

TC: Total cholesterol; TG: Triacylglycerol; FFA: Free fatty acid; ALT: Alanine aminotransferase; AST: Aspartate aminotransferase.
